# Exploring the factors impacting on access and acceptance of sexual and reproductive health services provided by adolescent-friendly health services in Nepal

**DOI:** 10.1371/journal.pone.0220855

**Published:** 2019-08-08

**Authors:** Pushpa Lata Pandey, Holly Seale, Husna Razee

**Affiliations:** School of Public Health and Community Medicine, University of New South Wales, Sydney, Australia; Università degli Studi di Perugia, ITALY

## Abstract

Adolescent-friendly health programs have been in place in Nepal since 2008, yet uptake of the services for sexual and reproductive health remains suboptimal. For uptake of these services to improve, a rich understanding is needed of the factors impacting their acceptance and utilization from the perspectives of adolescents, health care staff, and key community informants. This study applied a qualitative research design involving six focus groups with 52 adolescents and in-depth interviews with 16 adolescents, 13 key informants, and 9 health care providers from six adolescent-friendly health facilities in Nepal. Thematic analysis was conducted for data analysis. The key themes identified as barriers include access issues due to travel, institutional health care barriers, perceived lack of privacy and confidentiality, and the unprofessional attitudes of staff towards the sexual health needs of adolescents. These themes are underpinned by gendered ideology and a moral framework around the sexual behavior of adolescents. Interview responses suggested that health care providers take a policing role in prescribing adolescents’ conformity to this moral framework in their delivery of reproductive health care and services. While physical access to health services may be problematic for some adolescents, this is not the priority issue. Attention needs to be given to increasing the capacity of health care providers to deliver services without imposing their own and socially sanctioned moral frameworks around adolescent sexual behavior. Such capacity building should include training that is experiential and emphasizes the importance of confidentiality and non-judgmental attitudes.

## Introduction

Adolescence is a unique period of physical, psychological, emotional, and social maturation from childhood to adulthood [1–3]. Adolescence covers the 10–19 years age range and divided into early (10–14 years) and late (15–19 years) adolescence [4]. There are 1.2 billion adolescents aged 10–19 years in the world, comprising of up to 16% of the world’s population [5, 6]); 340 million of these adolescents live in South Asia [2]. Compared to twenty years ago, most of these adolescents are healthier and more likely to attend school and delay entering the labor force or marry and have children; many others, however, remain vulnerable to sexual and reproductive health (SRH) risks, including early marriage, early and unwanted pregnancy, unsafe abortions, and sexually transmitted infections and HIV/AIDS. It is estimated that 21 million girls aged 15–19 years and two million girls aged under 15 years become pregnant each year; 16 million of the girls aged 15–19 years and around 2.5 million who are under 16 years give birth in low and middle-income countries [7]. The majority of these adolescent pregnancies are the result of an unmet need for contraception [8]—about half (49%) of adolescent pregnancies are unintended [8]. Early pregnancy and childbirth can result in pregnancy-related complications, which have become the leading cause of death among adolescent females aged 15–19 years [9].

In 2016, 27.1% of Nepalese adolescent girls aged 15–19 years were married, according to the Nepal Demographic and Health Survey [10], despite the legal age of marriage having been set at 20 years for both male and females [11]. Of those late-adolescent married girls, only 14.5% were using modern contraceptive methods, a figure that had been almost stagnant since 2011, despite contraceptive knowledge being virtually universal (99.9%) in this age group [10]. Further, 16.7% of adolescent women aged 15–19 years were either already mothers or pregnant with their first child [10]. Although many married adolescent girls would have liked to delay or prevent further childbearing, they were often unable to obtain contraceptives before their last pregnancy, an unmet need amounting to as high as 34.9% in the 15-19-year-old cohort in Nepal [12]. While premarital sex and extramarital sex remain culturally unacceptable in Nepal [13], several local studies have shown that unmarried adolescents are becoming more sexually active [14, 15] with reported percentages of sexually active unmarried adolescents varying from 27–35% [13, 15, 16]. Since national-level surveys collect data from married adolescents only, data from a large number of adolescents who are not married are excluded. And it is likely that contraceptive use would be even lower among unmarried adolescents.

Adolescents face numerous challenges to access and utilization of SRH services globally [17–21]. Some of these challenges include inadequate SRH information arising from lack of access to SRH education [22, 23], early marriage and pregnancy associated with adolescents’ having minimal or no voice in decision making in SRH matters [24], and poor quality SRH services where concerns about privacy and confidentiality are related to health care provider and health facility infrastructure [20, 21, 25]. These challenges are largely associated with complex social, environmental, cultural, economic, and psycho-social factors [26]. In Nepal, adolescents’ access to SRH information and communication is limited [27] because of the cultural belief that talking about sex and sexuality leads to increased premarital sex, or encourages promiscuity [28, 29]. Nepal’s school curriculum includes SRH education as an optional course for adolescents aged 15–16 years. However, this curriculum component is often not implemented largely because teachers, aware of the social mores around this topic, are reluctant to discuss sensitive topics such as sexuality [30]. Similarly, in health facilities, health care providers’ own moral frameworks determine when adolescents are old enough to access SRH services [31], which limits their access to SRH information and services.

The World Health Organization (WHO) has argued that making SRH services “adolescent-friendly” is key to improving access to SRH services for young people [32]. Adolescent-friendly health services (AFHS) are defined by the WHO as services that are accessible, acceptable, equitable, appropriate and effective for adolescents as outlined in the WHO’s guidebook for developing national AFHS quality standards [33]. In other words, these are health care services that don’t restrict adolescents but do guarantee confidentiality, treat adolescents with respect and without judgment, and are within easy reach of and affordable for adolescents [33]. To deliver adolescent-friendly health services, many countries have made efforts to incorporate these features into their health care services with the aim of improving the arrangement, provision, and quality of SRH services for adolescents [34–38]. AFHSs are meant to be attractive to young people and thereby increase adolescent uptake of SRH services [32, 33].

Nepal has been progressively implementing its adolescent-friendly health program since 2008 to address the concerns surrounding adolescent SRH. By 2017, the program had been implemented in 70 of 75 districts (1134 public health facilities) [39]. Despite rapid scaling up of AFHS, studies show utilization of SRH services has not changed. A study conducted in the Bhaktapur district of Nepal in 2015 showed that only 9.2% of the adolescent participants (n = 338) had utilized SRH services from the health facility [40]. A second study conducted in the same year by the United Nations Population Fund (UNFPA) in another 12 districts, which included 72 health facilities with AFHS, revealed that 80% of participating adolescents had never visited the health facilities [41].

This paper attempts to present an in-depth account of the barriers associated with adolescents’ access to SRH care from AFHSs in Nepal. We argue that institutional healthcare barriers and socio-cultural factors, including moral values, maybe hindering adolescents’ access to SRH services.

## Methods

### Study setting

The study took place in the Dhading district of Nepal, a semi-cosmopolitan district embracing rural and urban populations. To select the facilities, health service utilization data for the two years preceding the current study were collected and analyzed from a total of 26 AFHS. We also observed these facilities using the Nepal National Standards for AFHS [42]. Based on the findings from health service utilization data and observations, each of these 26 health facilities were then rated and assigned to one of three categories: ≥70% compliance = Good or performing well; 40–69% = Medium or needs some improvement; and ≤39% = Poor or needs considerable improvement. These categories were devised based on reference to Chandra-Mouli, Chatterjee [43]. In researching eight low and middle-income countries, Chandra-Mouli, Chatterjee [43] looked at the national quality standards and criteria for AFHS developed by each country and assessed the quality of health service provision depending on compliance with the required standards of quality, ranking each according to the three categories mentioned above. Two health facilities from each category, or a total of six facilities, were purposively selected for the current study.

### Study design

In designing the study, we followed a qualitative approach so as to gain an in-depth understanding of the barriers to accessing adolescent SRH services from the participants’ (adolescents, health care providers, and key informants) perspectives. We conducted interviews in the study’s natural setting [44], that is, where participants lived, studied, or worked (homes, schools, and health facilities). Our study was informed by a social constructionist stance based on the view that knowledge and reality are constructed through social interactions [45]. We drew on some of the principles of grounded theory, maintaining proximity to our data and flexibility as the study proceeded [46]. For example, at the end of each interview or focus group, a preliminary analysis of the data was undertaken by making note of concepts, ideas, and potential themes; changes were made to the data collection process as the research process unfolded guided by this preliminary analysis. In this way, the data analysis began with data collection, and throughout the collection period, we constantly analyzed and looked at the patterns and themes arising from the data.

### Participant recruitment

We used purposive sampling for selecting adolescents, key informants (KIs), and health care providers (HCPs) for in-depth interviews [47]. Maximum variation sampling [48] was also applied to gather a diversity of perspectives from the different demographic segments (age, sex, caste, and ethnicity) of each participant group. The perspectives were collected from the following three sets of participants:

- Adolescents (15–19 years), both male- and female-identifying, and school attending and non-attending adolescents, as well as from different socio-economic backgrounds and ethnic groups, i.e., Brahmin, Chhetries, Newar, Tamang, and a highly marginalized indigenous group, Chepangs, were also included in the study. To facilitate the recruitment of adolescents, we displayed research flyers in schools and health facilities with the help of HCPs and schoolteachers. Tamang and Chepang adolescents were recruited with the help of a local female community health volunteer (FCHV) in the Tamang community and local Chepang leaders, who passed on the research information and our contact details to adolescents in their communities. We invited the adolescents who then contacted us to participate in the study.
- KIs were drawn from schoolteachers, health facility operation and management committees (HFOMCs), FCHVs, non-government organizations (NGOs), and government employees. KIs were notified about the research with the support of HCPs and local NGOs and through the distribution of research flyers. Snowball sampling was also used to recruit KIs [48].
- HCPs, both male- and female-identifying, were recruited from the six facilities selected as study sites. Only those HCPs who had received government-sponsored adolescent sexual and reproductive health (ASRH) program orientation, and who had been working in the selected health facility for at least six months were recruited as research participants.

Participant selection continued until the researchers were satisfied that adequate data would be provided to respond to the research questions [49].

### Data collection

To collect data we used semi-structured in-depth interviews (IDIs) with adolescents, KIs and HCPs, and focus group discussions (FGDs) with adolescents. IDI and FGD guidelines were developed by drawing on the literature and WHO quality standards for AFHS [33]. The IDIs and FGDs for adolescents explored their challenges in accessing SRH services from AFHSs. In interviews with HCPs, we explored their attitudes towards and perceptions and experiences of providing SRH services to adolescents. With KIs, we similarly explored their perceptions and attitudes regarding AFHS and existing socio-cultural beliefs and understandings about the SRH of adolescents. The IDI and FGD guides were translated into the Nepali language. Given the sensitive nature of the study’s topic of SRH within the Nepalese context, we developed vignettes to use as triggers for the interviews and FGDs. Vignettes are used to elicit information on sensitive topics when participants are uncomfortable talking about their own experiences and instead are encouraged to discuss the situation of the vignette character [50, 51]. In this study, participants were told a story about a 15-year-old girl and a 19-year-old boy who were in love and wanted to have sex with each other. In their responses, adolescents were asked to think about what information and support this couple might need, where they could seek support, and the barriers and challenges the couple might face.

The interviews and FGDs were conducted in venues chosen by participants that offered privacy and allowed free and open communication. We trained a male interviewer to conduct the interviews and FGDs in cases where participants were reluctant to share information with the female researcher and first author, PLP. The first author is a Nepalese who has worked in the area of SRH for more than 12 years. She also has played a major role in the design, implementation, and policy development around AFHS. All data collection was undertaken by PLP, except for one IDI and two FGDs. All interviews and FGDs were audio-recorded, and the main points noted. IDIs lasted between 35 minutes and 1.15 hours, on average, and FGDs between 2–2:30 hours. FGDs were held separately for male and female adolescents to encourage free and open expression about sexual health matters.

### Data management and analysis

The first author, PLP, who is fluent in both English and Nepali, transcribed the audiotapes verbatim from Nepali to English directly. Prior to transcribing all interviews into English, however, we first transcribed three interviews in Nepali, and PLP then translated these into English. The English language transcripts were then back-translated by an independent person, whose translation was compared with the original Nepali transcript. This process ensured that no major differences were evident in the translation. All transcripts were then saved on a password-protected computer accessible only to the researchers.

Trustworthiness [52] of the data was ensured by employing i) methodological triangulation by conducting IDIs and FGDs, ii) data source triangulation by interviewing different groups of participants (adolescents, health care providers and key informants), iii) member checking by clarifying the information obtained from the participants through summarizing and obtaining their validation during the interview itself, and iv) the involvement of the senior author, HR, who, as an experienced qualitative researcher, ensured confirmability by reviewing the coding of the IDI and FGD transcripts.

We used NVivo 11 for data coding and management. Data were then thematically analyzed using an inductive approach [53] and drawing on principles of grounded theory, as explained earlier. Preliminary data analysis continued throughout the data collection process to help identify further areas for exploration contributing to answering the research questions comprehensively [54]. Final and systematic data analysis was undertaken once all data collection was completed. In analyzing the data we followed the steps proposed by Braun and Clarke [55], i.e. transcription of interviews, familiarization by reading and re-reading during verbatim transcribing and looking for meanings and patterns for coding in the data, generating initial codes, generating and reviewing themes, defining and naming themes, and finalizing the analysis. PLP developed the initial codes which were then discussed with HR to reach consensus. A codebook compiled during this process listed the codes and their definitions, which were revised as coding progressed and new codes were added. Next, the codes were refined through a “deeper level of analysis” [56] to capture the implicit meaning behind what the participants were saying at the surface level of the data. During this part of the analytic process, we found that a moral framework seemed to underlie what the participants expressed on the surface. Codes within and across participants groups were reviewed for patterns, and these were further reviewed and developed into themes to answer the research questions [43]. During this process, PLP and HR discussed the themes at length to reach an agreement about them and whether they coherently represented the data.

### Ethical consideration

Ethical clearance for the study was received from the University of New South Wales (UNSW) Human Research Ethics Committee (HREC), Sydney, Australia, and from the Nepal Health Research Council (NHRC).

The SRH of young people is a highly sensitive research subject. Under such circumstances, it is deemed ethically acceptable to waive parental consent [57] when young people aged 15–19 years are: a) accessing health services independently; b) are cognitively mature to provide informed consent; and c) where parental involvement poses a risk to young people's emotional/mental/physical well-being [58]. According to Stablein and Jacobs [59], assessment of levels of maturity in adolescents is a professional judgment about minors’ competency, usually based on:

- Participants’ ability to understand the objectives and requirements of the research and other important considerations such as the voluntary nature of participation, and the potential risks and benefits;
- Participants’ ability to address the problem according to their own judgmental capacity; and
- Participants having the capacity to comply with the requirements of the research [59].

Further, the guidelines developed by Santelli et al. [60] for adolescent health research clearly state that adolescents aged 14 years and older possess the cognitive ability to decide on research participation similarly to adults. Waiving of parental consent was approved by both UNSW- HREC and NHRC. Informed consent was obtained from all the participants, who were assured of the confidentiality of their information. Pseudonyms have been used throughout to protect participants’ identities.

## Results

A total of 68 adolescents participated in the study: 52 of them took part in six FGDs (three male groups and three female groups); and 16 adolescents participated in IDIs. IDIs were also held with 13 KIs and 9 HCPs. All in all, the study had 43 female and 47 male participants (Table 1). The findings are presented according to the themes generated, reflecting barriers to accessing SRH services.

**Table 1:** Sample sizes: In-depth interviews and FGD participants.

Data collection methods	Type of respondents	Female	Male	Total
**In-depth interview**	Adolescents	6	10	16
	Health care providers	4	5	9
	Key informants	8	5	13
**Focus group discussion**	Adolescents 3 FGDs with female adolescents 3 FGDs with male adolescents	25	27	52
**Total**		43	47	90

### Distance to the health facility

Interviews and FGDs with adolescents, HCPs, and KIs clearly revealed that distance to the nearest health facility was one of the major barriers to utilizing AFHSs. This was highlighted in the FGDs—adolescents would go to a private pharmacy near their homes rather than spend the time required to get to the health facility in their village.

*It takes one hour for me to reach the health facility in my village*. *So*, *I go to a private pharmacy near my home*. (Male, FGD participant)

*This health facility is accessible to only wards 1*, *2*, *3 and 4*. *We cannot reach adolescents of other wards from this facility*. *Most of them from other wards go to the facility of the adjoining village*. *Others who have resources [money] go to Kathmandu*. (Shiva, male, HCP)

Some adolescents mentioned accessing SRH services when the facilities were close to their school or home. The location of an AFHS within easy physical distance is, hence, a likely factor in adolescents’ use of the services.

### Health care providers’ characteristics

The responses of both adolescents and HCPs highlighted a number of HCP-related characteristics contributing to the poor utilization of AFHSs. Age and gender of HCPs and their poor attitudes towards the needs of adolescents were some of the key areas that affected adolescents’ visiting AFHSs. Adolescents felt that HCPs who were much older than they were treated them like children and were unable to understand their SRH issues.

*Health workers are of the age of our mothers*. *We do not know a lot of things [SRH]*. *They should understand the issues and give proper advice*, *but they start to give a lecture*. (Male, FGD participant)

Adolescents’ experiences seemed to suggest that younger HCPs were more understanding and perhaps less judgmental of their problems, having more recently been through similar experiences.

*I have a friend who has completed a course on health*. *He belongs to my age*, *and he is working in the health facility*. *So*, *I ask him if I have any query*. *He can understand the issue of my age*. *He knows it all*, *so it is comfortable seeking advice from him*. *It is easy to talk to people of your age*. (Indra, male, 19 years)

While it is likely that being friends with the HCP may play a role, Indra’s use of the words “*it is easy to talk to people of your age*” clearly suggests the importance of a closer practitioner-adolescent client age difference.

Concerns about the age of the HCP were not only shared among adolescents. HCPs were themselves aware of the discomfort that adolescents felt during consultations with them; often, adolescents regarded much older HCPs as senior guardians and would, therefore, hesitate to share their SRH issues. For example, HCP Krishna mentioned:

*They [adolescents] see HCPs [of more than 30 years’ age difference] as guardian to them*, *so they cannot share their problems openly*. (Krishna, male, HCP)

Both male and female adolescents commonly experienced apprehension about the gender of the HCP, as the following quotes reflect.

*It is uncomfortable for male adolescents to share a problem with female staffs and female adolescents with male staffs*. (Mira, female, 15 years)

*It is difficult to show [genitals] when there is the presence of nurses [female staff]*. *The male HCP should be present there*. (Sam, male, 15 years)

HCPs noted that gender was a concern even when adolescents were consulting them about non-SRH related issues.

*If female adolescents have any issue*, *they often do not tell me when I am in the clinic*. *They would tell it to my wife and then my wife would ask me what to do*. *I would then give the advice accordingly*. (Ram, male, HCP)

In addition to age and gender, adolescents said that the HCP’s attitude was an important factor in their decisions to access SRH services from AFHSs. In the focus groups, there were heated discussions when adolescents were asked about their treatment by HCPs; voices were raised when they spoke about the “condescending attitudes” of the HCPs which had often prevented them from visiting AFHS for SRH services. Speaking of his experiences, Indra angrily stated:

*Health facility does not provide us with contraceptives such as condom when we are in need*. *Instead*, *they [HCP] show attitude*, *anger and speak to us harshly when we request condoms*, *which makes it difficult for us to access SRH services*. (Indra, male, 19 years)

Like Indra, most of the unmarried adolescents who sought SRH services had had negative experiences. For example, Maya, a 16-year-old female adolescent, reported that “*HCPs would say contraceptive is of no use to unmarried adolescents*, *and they should not use it before marriage*.*”*

The following explanation from Ram clearly shows his hesitation about providing SRH services to unmarried adolescents. The likely source of this reluctance is the deeply rooted socio-cultural norms and beliefs around adolescent sexuality and sexual behavior which perhaps result in HCPs making moral judgments about those who engage in premarital sex.

*There is some difference in how I deal with married and unmarried adolescents*. *If married adolescents come*, *I could easily provide counselling services and tell about contraceptives*. *But if unmarried adolescent comes*, *I would myself feel like why this adolescent has come here*, *and I wish this person would not use hormonal contraceptives as it might have side effects*. *I feel they should not use family planning before marriage*. (Ram, male, HCP)

Negative attitudes towards unmarried adolescents were expressed by both male and female HCPs, and likely served to prevent HCPs promoting SRH of unmarried adolescents. Moreover, their judgmental attitudes represent a barrier to adolescents seeking their help.

*Unmarried adolescents do not understand*. *They are young and have no idea of what could happen when they do this or that*. *They do so many things [sex] hiding from the parents*. (Sara, female, HCP)

Sara’s views are unsurprising given that even now, it remains unacceptable in Nepalese society for unmarried adolescents to be sexually active. Sara’s emphasis on *“unmarried adolescents do not understand*,” the way she spoke, and her facial expression all indicated her stern disapproval of their engaging in sexual activity.

The poor professional attitudes of HCPs may partly be shaped by their misunderstanding of what “adolescent-friendly” health service means. Asked about the procedures and requirements of providing AFHS, their responses suggested a poor understanding of the AFHS concept. Yet all the HCPs interviewed had attended ASRH training provided by the Ministry of Health and Population and should, therefore, have possessed specific knowledge about providing SRH services to adolescents.

Some HCPs did not know whether the AFHS was intended to be an ongoing or one-time program, or if it was an NGO-supported program.

*This program is lost for almost three years in our health facility*. *I do not know if it is a Government of Nepal program or it was supported by some NGO/INGO [temporary program]*. *Or if that particular NGO/INGO phased out*. *We are neither asked for any report nor are we providing it to the district*. (Sarala, female, HCP)

HCPs in this study had received only an initial orientation to the program three years previously; their poor understanding was, therefore, likely due to the absence of any regular follow-up training or reporting since then.

Another contributor to poor HCP attitudes towards SRH services for adolescents could be the frequent transfer of health facility staff. In Nepal, it is not unusual for HCPs working in one health facility to be transferred to another, either in the same district or elsewhere in the country [61]. When these transfers occur, formal handovers are not usual practice, and this can result in confusion about responsibilities for ensuring adolescent-friendly health services at the new place of appointment. For example, HCP Shiva, already working in an AFHS, was transferred to another facility which was also an AFHS. It might be assumed that, in this case, the HCP would be aware of his role in providing SRH services to adolescents. However, as there was no handover, Shiva stated that he was unsure of his accountability for AFHS in his new position. Interestingly, this situation seems to be peculiar to Nepal, where HCPs apparently feel that they need to be retrained in each new facility they go to, whether or not they already have the relevant training. In Shiva’s case, the absence of further training at his new place of work meant that he was not sure about his responsibility for AFHS. Thus, these circumstances can affect how some HCPs might treat adolescents who come to seek SRH services.

### Institutional health care barriers

Shortage of staff, SRH supplies, and medicine in health facilities and overburdened HCPs were institutional health care barriers revealed in this study. These barriers impacted HCPs’ capacity to provide SRH services to adolescents. Most of the HCPs noted that they were overtaxed with several responsibilities, including administrative work. Consequently, they had limited time to provide services to adolescents.

One of the adolescents indicated that busy HCPs provided a hasty service, which discouraged her from utilizing the AFHS.

*They are present every time I go there*. *However*, *they are normally very busy and do not have time to talk to us unless we are ill*. (Maya, female, 15 years)

In Nepal, the upgrading of sub-health post (one paramedic) to health post (three paramedics) has been ongoing since the fiscal year 2011–12. However, deficiencies remain in the deputation of staff in upgraded health facilities [62]. This may have resulted in upgraded facility staff being overburdened since the health post has additional responsibilities.

Similarly, shortages of SRH supplies and medicines also contributed to poor access. The unavailability of stocks of these items was a frequently related experience. As noted by interview participant Muna, then a 19-year-old female adolescent, except for medicines for fever, they had “*to buy all other medicines from [private pharmacy]*”.

According to the Department of Health Service, Nepal [62], 14% of family planning commodities, and 34% of essential drugs are routinely out of stock in health facilities throughout the country. Therefore, adolescents commonly have to buy medicines from pharmacies. Typically, adolescents do not have the money to pay for contraceptives and other requisite SRH supplies, so it may be expected that they will not be motivated to visit AFHS.

### Lack of privacy and confidentiality

Almost all of the adolescents, KIs and HCPs noted that lack of privacy and confidentiality was a major reason for adolescents’ reluctance to utilize AFHSs. Privacy and confidentiality were related to both the location and physical layout of the facility as well as to HCP attitudes. Adolescents perceived that neither the location of their local health facility nor its consultation area provided the visual privacy they expected.

*The health facility is open from everywhere*. *If there are some issues with private body parts*, *we feel shame to show those [body parts] as others might see*. (Female, FGD participant)

*There is only one examination room*. *Sometimes males enter when we are talking to the HCP*. *Sometimes male may be present in the room when a female’s check-up is taking place or vice-versa*. *This makes it very uneasy to share the problem*. (Mira, female, 15 years)

Both male and female adolescents seemed cautious of seeking help from HCPs who, often, lived in the same community, fearing breaches of their confidentiality. Interestingly, some of the adolescents said they did not want their cases to be discussed even among the HCPs themselves. Aarati’s and Indra’s comments showed how important confidentiality was to adolescents.

*Some of these issues need to be kept a secret between the HCP and us*. *I like HCP not sharing my visit to anyone*. *But they normally discuss our matters among them*. *I wish they would not do that and keep our issues confidential*. (Aarati, female, 16 years)

*Most HCP are socially known person in the community*, *and they know about our home and family*. *Moreover*, *we are afraid that they might share with our parents and our parents will scold us if they know we go to the health facility*. (Indra, male, 19 years)

Almost all adolescents perceived that there was a lack of confidentiality. Moreover, some adolescents had firsthand experience of their confidentiality being breached, as clearly noted by Nita, a 16-year-old female adolescent.

*I heard when the private [SRH] issues were shared outside*. *My aunt is the best friend of the one of the HCP*. *I heard this HCP talking to my aunt who is not a HCP*. *Even if they have some close friends*, *they should not be sharing the private matter of another person in the health facility*. *Such type of subject matter usually spread fast in the community from one to another*. (Nita, female, 16 years)

Adolescents’ privacy and confidentiality concerns as a barrier to utilizing SRH services from AFHSs were echoed by HCPs.

*Many [adolescents] might not come here because they are not confident and might have doubt that when they come here for services*, *their issues are not kept confidential*. (Kiran, female, HCP)

Kiran’s comment suggests that HCPs were not trusted to keep things confidential. But this was not the only issue; Ram, an HCP, noted that adolescents did not seek SRH services from him because he was a member of their local community. This is more of a concern in rural areas, where communities are close-knit, and most people know each other. Under those circumstances, adolescents may not want a member of their own community, knowing if they were engaging in socially unacceptable sexual activity. This is discussed in more detail under the later theme, *Socio-cultural norms and attitudes towards adolescent SRH in the community*.

### Lack of information on SRH

Interviews within all participant groups revealed that information on SRH for adolescents in the communities was scarce and, therefore, adolescents were uninformed about potential reasons for seeking SRH services. All participants spoke of the difficulty of talking about sex and sexual relations openly, a situation that affords adolescents very little opportunity to learn about SRH either at home or in school. As schoolteacher Tek noted, *“Our culture*, *religion*, *and tradition do not allow talking openly about sex and sexual matters*.*”*

Krishna, a male HCP, said that *“families are not habitual in talking about (sex)*,*”* highlighting that, generally, *“the practice of sharing or talking about reproductive health in front of family or father or brother is absent in our society*.” These responses suggest the existence of a “culture of silence” on the topic of SRH and may be a result of the moral values within Nepalese society.

Adolescents, too, noted how difficult it was for teachers to discuss SRH topics; when teachers were required to conduct SRH lessons, they would skip or rush through some topics, providing little opportunity for discussion. This was also observed by key informants, as reflected in the following quote.

*Whenever teachers had to teach about sexual health or bodily changes*, *they ask students to study themselves*, *and they would go out of the room*. (Nirmaya, female, KI)

Teachers found it even more difficult to teach SRH topics if they belonged to the same community as the adolescents since there was the likelihood that “*one of the teacher’s daughter*, *son or sister will be in the same class*.*”*

The implications of the culture of silence surrounding SRH include not only poor sexual health literacy but also the high likelihood of adolescents having poor knowledge of SRH services offered by AFHSs.

### Socio-cultural norms and attitudes towards adolescent SRH in the community

Throughout the interviews with adolescents, key informants, and HCPs, as well as in FGDs with adolescents, a consistent and recurring theme involved the underlying socio-cultural norms and attitudes towards adolescents’ sexual behavior. It is clear from our findings that prevailing norms were a major factor influencing adolescents’ poor utilization of AFHSs. Any sexual behaviors were considered, especially for unmarried adolescents, to be “KHARAB BANI” which literally means “bad behavior.” Further, unsanctioned sexual activity was judged to be “BIKRITI” or a “bad influence” on other adolescents. *KHARAB BANI* and *BIKRITI* are quite derogatory terms in Nepalese society, and such labelling is reflective of a code of morality governing adolescent sexual behavior. The notion that sexual behavior among unmarried adolescents is “bad” leads to stigmatization and rejection with their communities.

What is notable is that it was mostly adolescent girls who were subject to the *KHARAB BANI* or *BIKRITT* labels when participants spoke about this phenomenon. Moreover, it was implied that sexual behaviors were initiated by females and then spread through the community. This view can be explained by a predominantly gendered ideology of morality, which also serves as a form of social control over female sexuality and sexual behavior [63].

*It will be bad if their [female adolescents] character is wrong*. *For instance*, *if a girl has a sexual relationship and gets pregnant and could not get the baby aborted*, *her future will be ruined*. *Even if she tells about it to the boy whom she had a relation*, *he might not accept her*, *or it could be that she had a relationship with several boys*. (Lalit, male, KI)

Lalit’s concerns about an adolescent girl becoming pregnant, and the associated risks, as a result of her sexual engagement exemplify the gendered attitudes inherent in Nepalese society. His suggesting that *“it could be that she had a relationship with several boys”* reveals the persistence of the belief that female sexuality is to blame for what society deems *bad behavior*.

Deep-seated socio-cultural norms and attitudes around female sexuality may prevent adolescent girls seeking SRH services for fear of social stigma and labelling as a consequence of the sexual activity. Even male adolescents were afraid of bringing shame to their families if they visited AFHS for SRH care, as Raj, a 19-year-old male adolescent, makes explicit:

*If I visit the health facility and if this information reaches from HCP to my villagers*, *then it is a matter of shyness in public*. *My parents will get embarrassed*, *and in total*, *if all society knows this information*, *they will make gossip*. (Raj, male, 19 years)

## Discussion

This study provides insights into some of the challenges faced by adolescents in accessing SRH services from AFHSs in Nepal. The findings from this study suggest that health care providers’ characteristics, institutional health care barriers, lack of privacy and confidentiality, lack of information on SRH and socio-cultural norms and attitudes relating to adolescents’ SRH in the community were key factors contributing to the utilization of SRH services by adolescents.

In our study, institutional health care barriers reported by adolescents included shortages of ASRH-trained HCPs and unreliable supply of medicines and other items. The shortage of ASRH-trained HCPs in Nepal’s AFHSs has been previously reported [41], and often results in staff being overburdened with responsibilities and the allocation of non ASRH-trained HCPs, compromising the quality of health care provided to adolescents. Due to this issue, it is important that training is provided to all health care providers in ASRH across the country to maintain the quality of ASRH services in health facilities. Adolescents were also unwilling to visit health facilities because of the poor availability of SRH items and other regular medicines, which are free of cost, but which are often out of stock. This requires them to purchase supplies from private pharmacies. According to other studies in Nepal, adolescents have failed to access SRH services from health facilities (i.e. obtain contraceptives and other medical supplies), because of the adolescents’ financial inability to purchase the recommended medicines [41, 64]. In a study conducted in Ethiopia, adolescents who received drugs during their visit to the health facility were 2.7 times more satisfied and willing to seek services than those who did not [65]. Hence, the reliable and regular availability of sexual health and medical supplies from health facilities seems increasingly necessary.

A key theme developed in this study was the lack of information on SRH for adolescents. HCPs found it challenging to communicate and disseminate information on SRH to unmarried adolescents, especially to those who lived in the practitioner’s community. Some HCPs appeared to be quick to judge unmarried adolescents, and their attitudes were mirrored in their treatment of adolescents who came to the facility. Indra and Maya experienced HCPs’ anger when they went to the health facility to get condoms; others spoke of being “lectured at” for seeking SRH services. It could be argued that displaying this kind of attitude is a form of “moral policing,” whereby HCPs appear unfriendly or judgmental towards adolescents—or at least towards unmarried adolescents, or married adolescents wanting an abortion. We argue that a moral framework seems to underpin the unprofessional attitudes of many HCPs.

This moral framework is constructed from the prevalent morality relating to premarital sexual activity in Nepalese society. As we noted at the beginning of this article, sexual activity among unmarried adolescents is unacceptable in Nepal [66, 67]. This is true for many other countries besides Nepal [19, 68, 69] and in these countries, too, a moral framework applies. For example, a qualitative study of 15-19-year-olds in Vanuatu noted that HCPs denied SRH services and that their disapproval of adolescent sexual experience was guided by their own moral values [19]. Similarly, a study conducted in South Africa among 28 nurses working in both rural and urban health facilities found that the nurses’ own value systems influenced their judgments about the character and motivation of adolescents who sought SRH services at health facilities [68]. Our findings clearly show the same circumstances at work in Nepal.

Foucault explains morality as a set of values or “rules of action” people are expected to follow as prescribed by various parties within society [70]. In our study, these parties included families, schools and religious institutions, as well as health facilities. HCPs such as Ram and Sara were clearly guided by the moral values they learnt through socialization within the Nepalese culture. The morality framework that HCPs seem to apply shapes how they deliver ASRH services. This was especially apparent in the case of older HCPs we interviewed for our study. When a significant practitioner-adolescent age gap existed, it was difficult, particularly for older HCPs, to accept adolescents being sexually active. Thus, while HCP Ram found it easy to talk about contraceptives to married adolescents, he questioned why unmarried adolescents needed contraceptives, showing the influence of his moral framework on his delivery of services to adolescents.

When adolescents deviated from this prevalent moral framework, they were subject to disparaging labels such as *KHARAB BANI* or *BIKRITI*. The purpose of derogatory labelling is to suppress behaviors that deviate from the socially acceptable, as Bob Fine [71] has noted; in the current study, the behavior is the sexual activity of unmarried adolescents. However, what was more apparent from our findings is the gendered ideology that the labelling expresses. Female adolescents were found to be more vulnerable to the “bad behavior” label, even though their male counterparts are, presumably, equal partners in the behavior. It has been the first author’s own experience of growing up and working in Nepal that this gendered ideology is commonly accepted. As in most South Asian societies [72], sexual behavior is a gendered phenomenon in Nepal, and sexual intercourse is predominantly socially sanctioned for females only within the boundary of marriage [73]. Yuval-Davis [74], in her book *Gender and Nation*, argues that in many countries, women are the bearers of honor in the family and community. In some countries, allegedly bringing dishonor has resulted in extreme consequences for women, such as the phenomenon of honor killings [75]. While there is no empirical evidence of honor killings in Nepal, there has been some media attention on similar kinds of consequences occurring recently [76, 77]. Connell [78] makes the point that premarital sex has few repercussions for men; rather, it is associated with men’s position in a patriarchal society. Connell’s statement is certainly true in the context of our study and is made explicit in the application of the derogatory *BIKRITI* label solely to female adolescents. Patriarchal attitudes are clearly evident in Nepalese society when adolescent boys are not blamed or judged in the same manner as girls and do not suffer the same consequences for engaging in premarital sex.

Within a society where premarital sexual activity is not socially sanctioned, for adolescents to access and use SRH facilities, they must have some level of trust in the HCPs. But as the results of this study suggest, the perceived lack of privacy and confidentiality experienced by Nepalese adolescents leads to distrust of health care providers. Trust is identified as an essential element in successful provider-patient relationships [79] and is one of the most important elements influencing adolescents’ willingness to seek care from health services [80, 81]. The ability to trust their local HCP is crucial for unmarried adolescents in a moral context where their sexual behavior needs to be kept private if they are to avoid unfortunate consequences. We found that the adolescents in our study feared the consequences of visiting the health facility for SRH services and having details of their consultation disclosed to their parents or other significant adults in their communities. The adolescents felt ashamed and fearful that their actions would bring shame to their families. Thus, trusting their HCPs to keep the details of their visit confidential, including, as respondents reported, not even sharing it with another HCP, is absolutely essential if adolescents are to utilize AFHSs.

While our study showed how inadequacies of the built environment and infrastructure of the health facility impact the privacy of adolescents, having trust in the HCPs was more important than the physical limitations. Literature has suggested that inadequate HCP capacity building can result in negative attitudes towards adolescents SRH [82, 83]. Examining their own values and learning appropriate communication skills needs to be a part of training for HCPs. Such training needs to be in an experiential and immersive format emphasizing professional ethics over personal moral frameworks in their provision of adolescent SRH services [84]. Value clarification has been successfully applied to reduce HIV-associated stigma [85], and to improve medical abortion care [84, 86, 87].

## Limitations and strengths

Our study has several limitations. Owing to constraints of time and resources, we chose six AFHSs that were in geographically accessible locations in Nepal. However, the choice of facilities within relatively easy reach meant that the geographical barriers to SRH services faced by adolescents living in more remote locations were not adequately reflected in our analysis. The challenges for geographically isolated adolescents would likely be different from those reported by adolescents living in the catchment areas of the six selected research sites.

Further, we selected participants from the ethnic groups represented in the district, including Chepangs, a highly marginalized indigenous group. While we felt their inclusion to be important, we were able to conduct only one interview and two focus group discussions with Chepang adolescents. The resulting data was inadequate to identify distinctive patterns within this group. This was also the case with other caste groups; the number of participants we were able to recruit within the constraints of time and resources, did not yield adequate data to observe patterns among these different groups. While we made extra efforts to include marginalized indigenous groups, our study did not include participants from Muslim or Christian minorities, and therefore, their voices are not reflected in this study. Similarly, the study did not include adolescent participants with different sexual orientations and gender identity including transgender. The inclusion of these groups in future studies would help illuminate more diverse perceptions and experiences of adolescents, provide us with richer insights and a more nuanced picture of what contributes to the utilization of adolescent-friendly health services, particularly the cultural and social context in which adolescents seek these services in Nepal.

The primary researcher (PLP) in this study is a Nepali woman who is familiar with the socio-cultural context shared by its participants, which may have influenced how some participants responded to the questions. Some HCPs and KIs may have also omitted some information from their responses because they expected a researcher with this background to know the Nepalese context. For instance, some of the participants would say *“you know it*, *Didi (sister-primary researcher)*,” assuming that PLP already knew the answer and so not providing any further detail. It is also possible that some HCPs may have given the answers they thought this researcher would want to hear. To minimize this type of researcher influence, PLP used probes to explore participants’ answers. Moreover, PLP had no close connection with the participants in this study and did not know any of them personally. Thus, it is unlikely that PLP’s Nepalese background would have influenced the data collection process negatively. It is more likely that her Nepalese background facilitated a deeper and more comprehensive understanding of the data.

Notwithstanding the above limitations, the study has numerous strengths. PLP’s Nepali background enabled her to develop rapport and trust among study participants, which has strengthened the trustworthiness and credibility of the data collected [88]. Using creative methods of data collection such as vignettes and drawing exercises elicited rich information, helping us to gain in-depth insights into the experiences of adolescents seeking SRH services in Nepal.

## Conclusion

Adolescent-friendly health services may conceptually be the ideal way of providing effective SRH services to adolescents. However, as this study has shown, their implementation requires revisiting and rethinking what “adolescent-friendly” means within the Nepali context, particularly from the viewpoint of the socio-cultural setting. While the WHO’s guidebook for developing national quality standards for AFHS gives emphasis to the physical structure of facilities, their geographical location, the cost of services to adolescents, and ready availability of supplies, this study has raised the question of how “friendly” these facilities truly are for adolescents living in a society which views adolescent sexual behavior through a moral lens. Services provided can be physically accessible and affordable to adolescents, but unless the moral framework of those providing the services changes, these services are unlikely to be fully utilized by adolescents. Thus, there is a need to address not only the structural components of these facilities, but also build the capacity of health care providers to set aside their own moral values in favor of professional practices that put the needs of the adolescents first without judgment and in a manner that develops trust in them and the services they provide. At the same time, it is essential to involve whole communities and policymakers in raising awareness of the gendered nature of the prevailing ideology underpinning the moral framework around adolescent sexual behavior.
